# A tourism dataset from historical transaction for recommender systems

**DOI:** 10.1016/j.dib.2023.109990

**Published:** 2023-12-19

**Authors:** Choirul Huda, Yaya Heryadi, Lukas, Widodo Budiharto

**Affiliations:** a Computer Science Department, Bina Nusantara University, Jakarta 11480, Indonesia; b Cognitive Engineering Research Group (CERG), Universitas Katolik Indonesia Atma Jaya, Jakarta 12930, Indonesia

**Keywords:** Tourism dataset, Recommender system, Historical transaction, Item, User, Rating, Collaborative filtering

## Abstract

The tourism industry has currently grown in various aspects, including the types of attractions, their quantity, and the number of tourist visits in various regions, contributing positively to both regional and global economies. Historical transactions are essential for developing recommender systems, utilizing techniques such as Collaborative Filtering and Demographic Filtering. TripAdvisor is a reputable website providing a wide range of accessible tourism information, including attractions, user profiles, and ratings. However, this unstructured raw data requires processing to create an adequate dataset for recommender systems. This study conducted a series of data processing steps on the raw data, including data restructuring, validation, content addition, integration with Google Maps, normalization, and modeling. This study successfully produced an original dataset comprising User Transaction, Item or Attraction, Attraction Type, Continent, Region, Country, City, and Visiting Mode. It also includes an entity relational model for tourism in Indonesia, particularly in Bali, Malang, and Yogyakarta regions, based on various global user experiences. This dataset is adequate and essential for developing various models of tourism recommender systems such as using Collaborative Filtering.

Specifications Table

**Table:** 

Subject	Computer Science, Tourism.
Specific subject area	This dataset contains various attributes indispensable for the research and development of recommender systems in the tourism industry sector. Various techniques in machine learning can be implemented using this dataset.
Data format	Restructured, Filtered, Validated, Analysed.
Type of data	Nine Tables (Excel format) consisting of historical transactions for tourism activities with additional information about location, visiting mode, and attraction types.
Data collection	Unstructured information from the TripAdvisor website was collected using the WebHarvy crawler module, resulting in a single, unnormalized table in Excel format. This crawler module was also applied in a previous study [1]. To produce the structured data equipped with relational data model consisting of nine tables in the dataset, the researchers analysed, restructured, filtered, validated, incorporated Google Maps searching, normalized, and encoded the data. The targeted transactions consist of tourism experiences in Indonesia, especially in Bali, Malang, and Yogyakarta regions from October 2022 to January 2023. The data is in English and contains some attributes indispensable for recommender systems [2]. Every region covers ten of the most popular attractions based on the number of visits. Data value validation was conducted to ensure the standardization such as a city name of the city; for examples, “NY”, “New Yok”, and “New York” were standardized to “New York”, while for a country name, such as “zÃ¼rich” was corrected to “Zurich”. A normalization process was conducted to ensure that every table is a unique object and has unique attributes with no partial dependency and no transitive dependency on it. Additional attributes were generated for every table to uniquely identify tuples as primary keys with foreign keys needed for references to the other tables. Google Maps was used to search the additional data regarding the location of users namely cities, countries, regions, and continents based on users’ city. The transaction was selected through the exclusion of unknown data such as visit mode, visit year, and visit month.
Data source location	Tourism activities were generated from the TripAdvisor website with additional information about user locations obtained from searching through Google Maps.
Data accessibility	Repository name: Mendeley Data Data identification number: 10.17632/h58s544674.1 Direct URL to data: https://data.mendeley.com/datasets/h58s544674/1 Instructions for accessing these data: open access through the URL or DOI for non-commercial purposes.

## 1 Value of the Data

• The data values encompass various functionalities and aspects of tourism, including users, items (tourism attractions), ratings, and visiting attributes, all of which are essential inputs for studies in recommender systems.
• To facilitate research on recommender systems using Machine Learning techniques, the dataset was normalized and selectively includes data from the last 10 years. Additionally, it features only the top 10 most popular items from each region in Bali, Malang, and Yogyakarta.
• This dataset is valuable for researchers in the field of Artificial Intelligence (AI), specifically for conducting research in tourism recommender systems utilizing certain techniques in Machine Learning (ML).
• The data values are logically interconnected, building a comprehensive perspective of tourism visits in Indonesia's Bali, Malang, and Yogyakarta regions. The relational keys within the dataset facilitate researchers' understanding and utilization.

## 2 Data Description

**Fig. 1:**
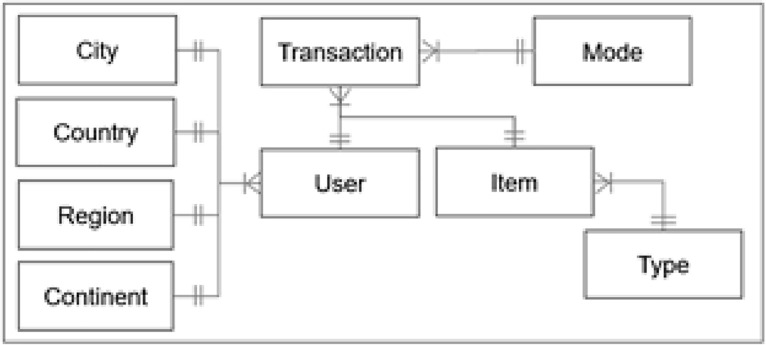
Entity relationship diagram of the dataset.

The tourism industry has currently grown in various aspects, including the types of attractions, their quantity, and the number of tourist visits in various regions, contributing positively to both regional and global economies [3]. There is an urgent need for studies in this sector to enhance service quality through smart solutions like recommender systems, which offer more personalized tourism experiences [4]. The tourism dataset offers crucial data for experimental studies in tourism recommender systems. The source of the dataset was crawled from a reputable website namely TripAdvisor website in providing tourism historical transactions in Indonesia. This data source draws inspiration from previous studies [5,6] in tourism recommender systems. The raw dataset that was crawled from this website consists of some unnormalized attributes for the descriptions of users, items, and transactions (ratings), unstructured data format, and consist of sparse data values, so it couldn't be used for some techniques in recommender systems. Through some data processing for the raw dataset incorporated with the Google Maps search engine, this study has succeeded in providing the original tourism dataset for tourism recommender systems that functionally consists of 52,930 transactions, 33,530 users, and 30 items. The relationship between the entities in the dataset is presented in Fig. 1.

Fig. 1 facilitates the description of each entity, providing detailed information about every data piece in the dataset. The relationships within the dataset serve as a guideline for studies focused on implementing recommender system applications. Definitions for each column name in the normalized dataset are detailed from Table 1, Table 2, Table 3, Table 4, Table 5, Table 6, Table 7, Table 8, Table 9. Each user transaction is listed in separate rows, with every column displaying encoded values. Research in recommender systems utilizing this dataset can be executed using various machine learning techniques such as Collaborative Filtering [1,[7], [8], [9]], Content-Based Filtering [10], Demographic Filtering [1], Centex-Aware [2,11], and Hybrid Technique [1,5] for smart tourism solution [12].

**Table 1:** List of attributes of user table.

Column	Description
UserId	Manually generated user identity based on the original identity (key) to uniquely identify every user.
ContenentId	Manually generated continent identity to uniquely identify every continent.
RegionId	Manually generated region identity to uniquely identify every region.
CountryId	Manually generated country identity to uniquely identify every country.
CityId	Manually generated city identity to uniquely identify every city.

**Table 2:** List of attributes of transaction table.

Column	Description
TransactionId	Manually generated identity of transactions to uniquely identify every visiting transaction.
UserId	Manually generated user identity based on the original identity (key) to uniquely identify every user.
VisitYear	Visit year of users.
VisitMonth	Visit month of users.
VisitMode	Visit modes in which are 01 (Business), 02(Couples), 03(Family), 04(Friends), and 05(Solo).
AttractionId	Manually generated attraction identity to uniquely identify every attraction (item).
Rating	Rating values that were given by users on a scale of 1 to 5.

**Table 3:** List of attributes of continent table.

Column	Description
ContinentId	Manually generated continent identity to uniquely identify every continent.
ContinentName	The names of continents according to the regions. They were manually produced through Google Maps Search.

**Table 4:** List of attributes of region table.

Column	Description
RegionId	Manually generated region identity to uniquely identify every region.
RegionName	The names of regions according to the countries. They were manually produced through Google Maps Search.
ContinentId	Continent identity of the region.

**Table 5:** List of attributes of country table.

Column	Description
CountryId	Manually generated country identity to uniquely identify every country.
CountryName	The names of countries according to the crawled data. The data pre-processing was conducted for unknown countries or multiple values such as: “Sorocaba, SP” which has been transformed into 2 values in 2 columns which are “Brazil” for the value of countries, “Sorocaba” for the value of city based on Google Maps Search.
RegionId	Region identity of the country.

**Table 6:** List of attributes of city table.

Column	Description
CityId	Manually generated city identity to uniquely identify every city.
CityName	The name of cities according to the crawled data. The data pre-processing was conducted for unknown cities or multiple values such as: “Sorocaba, SP” which has been transformed into 2 values in 2 columns which are “Brazil” for country name and “Sorocaba” for city name based on Google Maps Search.
CountryId	Country identity of the city.

**Table 7:** List of attributes of mode table.

Column	Description
VisitModeId	Manually generated visiting mode identity to uniquely identify every visiting mode.
VisitMode	The mode of visit according to the crawled data. The data pre-processing was conducted for unknown visiting modes such as: “Oct 2022 • Couples” that have been transformed into 3 values in 3 columns in which are “Oct” for VisitMonth, “2022” for VisitYear, and “Couples” for VisitMode.

**Table 8:** List of attributes of item table.

Column	Description
AttractionId	Manually generated attraction identity to uniquely identify every attraction (item).
AttractionCityId	Manually generated attraction city identity to uniquely identify every attraction city.
AttractionTypeId	Manually generated attraction type identity to uniquely identify every attraction type.
Attraction	The attraction or item of tourism from the crawled data.
AttractionAddress	The location of attractions or items from the crawled data. The data normalization was conducted for unknown locations through Google Maps Search.

**Table 9:** List of attributes of type table.

Column	Description
AttractionTypeId	Manually generated attraction type identity to uniquely identify every attraction type.
AttractionType	The type of attraction is based on the crawled data. The data pre-processing was conducted for the unknown attraction type such as the type of attraction “Jogja Bay Waterpark” that has been successfully found it's attraction type which is “Water Parks” based on the value of the string “Waterpark” in columns of attractions together with the comparison of the similarity values from other attractions.

Table 1 presents user profiles regarding their geographic location produced by combining data sources from TripAdvisor and search results from Google Maps. The other attributes are collected from Google Maps manually through the user's country and the user's city from TripAdvisor.

Table 2 presents the past tourism activities of each user along with the time of occurrence, accompanied by the impressions of each user while visiting each attraction. The attributes offering geographic location information are generated using the same process as that for the User table. This data facilitates the analysis of user experience and interests, presenting opportunities for further research in the realm of recommender systems. The dataset encompasses a total of 52,930 transactions.

Table 3 describes the user's continent in terms of the user's geographic location. There are 5 continents provided in this dataset.

Table 4 describes the user's region in terms of the user's geographic location. There are 21 regions provided in this dataset.

Table 5 describes the user's country in terms of the user's geographic location. There are 164 countries provided in this dataset.

Table 6 describes the user's city in terms of the user's geographic location. There are 9,142 cities provided in this dataset.

Table 7 describes the user's mode of visit when using tourism products or attractions. Based on the results of data processing, five modes of visits were obtained: Business, Couples, Family, Friends, and Solo.

Table 8 describes the tourism products or attractions that have been visited by each tourist, which includes the attraction code, attraction name, type of attraction, and location of the attraction. There are 30 attractions provided in this dataset that are distributed in three areas in Indonesia: Bali, Malang, and Yogyakarta.

Table 9 describes the type of attractions that were visited by each tourist. The attraction types were obtained from the website of TripAdvisor as a data source. There are 17 attraction types provided in this dataset.

The transaction table, serving as a coordinating table in the relational data schema with other tables (entities), contains foreign keys that facilitate the retrieval of further information through join or merge processes with other entities. Utilizing this dataset allows for smoother soft computation compared to direct processing of the original, crawled data. The dataset consists of normalized data that has many perspectives for analysis. Table 1 provides some opportunities for study in recommender systems through some techniques in Machine Learning. Table 9 presents the aggregated number of visiting users by user's continents and visiting months. This dataset can also be utilized for analysis in the tourism industry, particularly in areas requiring greater attention, like product and service development, tailored to user demographics and visit timings. To ensure a pleasant tourism experience, it is vital to cater to various preferences, ensuring the provision of quality facilities and service [13].

## 3 Experimental Design, Materials and Methods

**Fig. 2:**
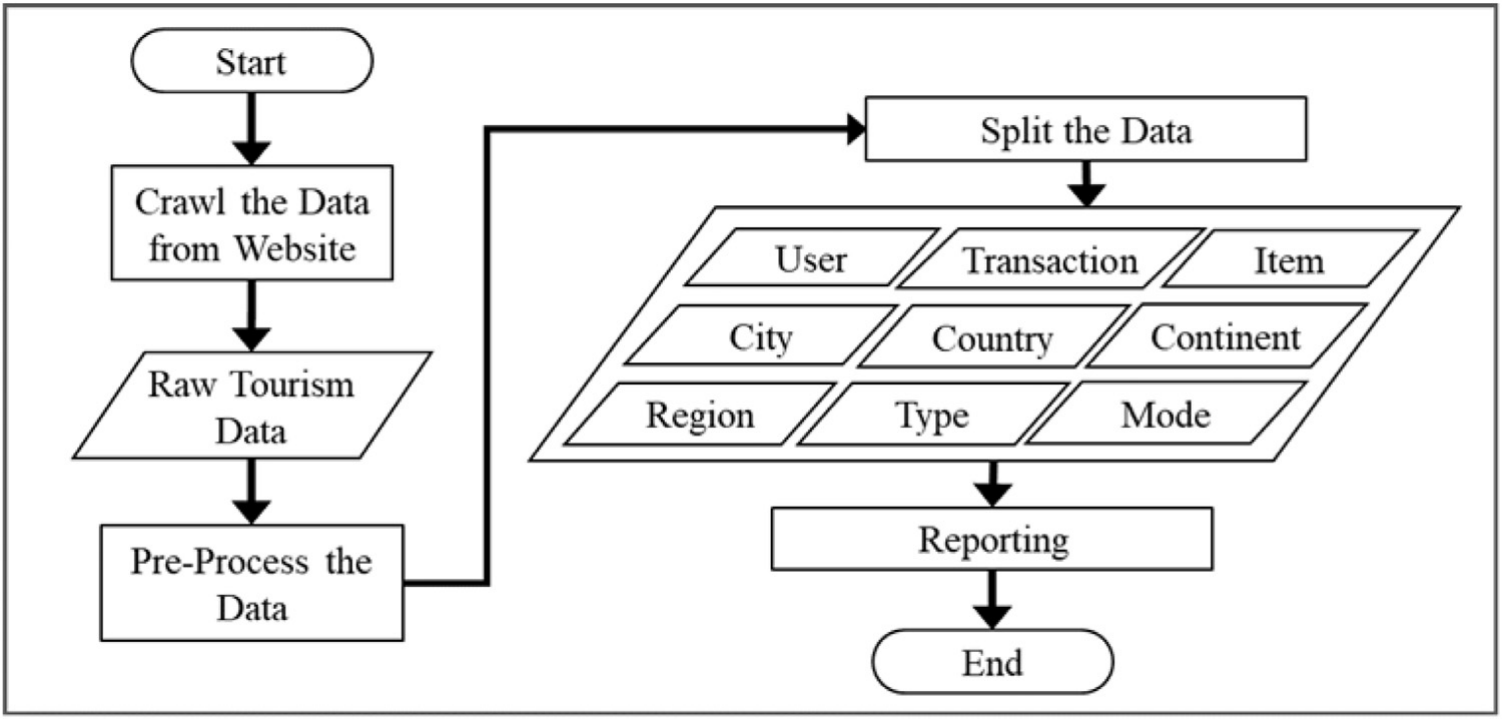
Activity flow of tourism dataset development.

The serial process through four main steps was conducted to produce a non-commercial tourism dataset in supporting valuable data for research purposes in a smart tourism industry through a tourism recommender systems development as presented in Fig. 2. Through the WebHarvy crawler module, the unstructured single table was produced from the TripAdvisor website. The following steps were conducted manually using Microsoft Excel incorporating Google Maps for user location search. Previous studies in dataset creation by [14], [15], [16] have been reviewed to build comprehensive knowledge in methodology and experimental design.

The TripAdvisor website provides essential information for recommender systems regarding the available information of users, items, ratings, and other transaction attributes in tourism activities. The next step involves crawling the data resulting in a single table in Excel format for tourism activities in Bali, Malang, and Yogyakarta regions. The crawled data, rich in attributes, requires thorough pre-processing to develop an adequate dataset suitable for recommender systems. This study conducted the following steps for the crawled raw data that has been stored in a single unnormal table:

• Selecting Data: selecting only the textual data from tourism transactions as candidates for the datasets.
• Removing Sparsity: Selecting only known data as candidates for the dataset, excluding missing values like unknown users, unknown items (attractions), and unknown transaction attributes such as user location, item location, visit time (month, year), and visit mode.
• Normalizing Data Values: Manually correcting unnormalized data in cells. For example, “Australind” and “Australian” were revised to “Australia” for country names, “zÃ¼rich” was corrected to “Zurich” for city names, and unknown attraction types were identified by finding similar types.
• Splitting Columns: Separating columns with multiple values into unique attributes, such as dividing a date column into VisitMonth, VisitYear, and VisitMode.
• Adding Columns: Enhancing data completeness by adding new columns like region and continent for user profile completeness. The values for these new columns were derived from Google Maps searches based on existing user address data, which may include city and country.
• Splitting the Table: Dividing the table into seven normalized tables: Transaction, User, Item, Type, Mode, City, and Country.
• Adding New Tables: Inserting additional tables for Region, Continent.
• Encoding Data Values: Converting data values into numeric types to facilitate smooth computations in various Machine Learning techniques.

**Fig. 3:**
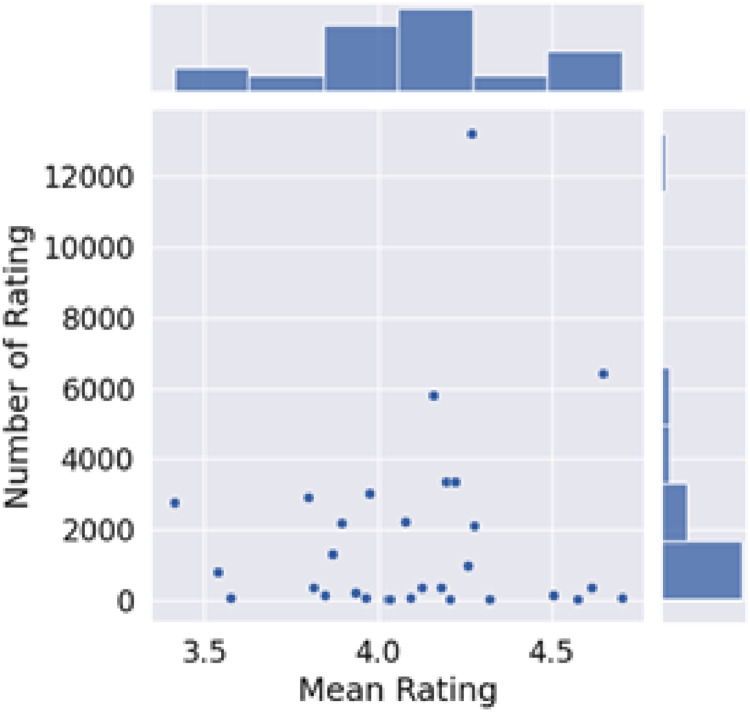
Distribution of mean ratings and number of ratings.

Fig. 3 visualizes the rating recap of the dataset through two perspectives which are the mean ratings and number of ratings according to the result of the dataset development process. A high-level interface for drawing informative statistical graphs for the dataset is provided through Seaborn, a Python-based data visualization library. This data visualization offers an overview of the average distribution of ratings for tourism products, based on the 52,930 transactions included in this dataset. It allows for diverse assessments of tourism user experiences, as reflected by the given average ratings. The dataset is ready to be used for studies in recommender systems through some techniques in machine learning.

## Limitations

While the dataset meets the requirements for use in research and development of a tourism recommendation system, its scope is limited to transactions from only three popular places in Indonesia: Bali, Malang, and Yogyakarta. This dataset still presents the ten most popular tourist attractions for each of these regions. In future developments, we propose to add several transactions for more regions and attractions.

## Ethics Statement

The purpose of this dataset is to provide useful data for research purposes, not for commercial use. WebHarvy crawling module was also conducted for collecting public tourism information in the previous study [1].The users' identity information from the dataset's source has been substituted with new and unique identifiers ensuring that the original identities remain inaccessible to the public . This article's dataset has been fully anonymized, and the platform's data redistribution policy has been complied with.

## CRediT authorship contribution statement

**Choirul Huda:** Conceptualization, Methodology, Data curation, Software. **Yaya Heryadi:** Writing – original draft, Supervision. **Lukas:** Validation, Writing – review & editing. **Widodo Budiharto:** Visualization, Investigation, Supervision.

## Data Availability

Tourism Dataset (Original data) (Mendeley Data)

## References

[1] Kbaier M.E.B.H., Masri H., Krichen S. (2018). A personalized hybrid tourism recommender system. Proc. IEEE/ACS Int. Conf. Comput. Syst. Appl. AICCSA.

[2] Ricci F., Rokach L., Shapira B. (2015).

[3] Tourism W., Unwto O. (2020). UNWTO World Tourism Barometer and Statistical Annex, May 2020. UNWTO World Tour. Barom..

[4] Gretzel U., Sigala M., Xiang Z., Koo C. (2015). Smart tourism: foundations and developments. Electron. Mark..

[5] Ravi L. (2019). Hybrid location-based recommender system for mobility and travel planning. Mob. Networks Appl..

[6] Abbasi-Moud Z., Vahdat-Nejad H., Sadri J. (2021). Tourism recommendation system based on semantic clustering and sentiment analysis. Expert Syst. Appl..

[7] Gao L., Wu J., Zhou C., Hu Y. (2017). 31st AAAI Conf. Artif. Intell. AAAI 2017.

[8] Choi I. (2019). A recommender system based on personal constraints for smart tourism city*. Asia Pac. J. Tour. Res..

[9] Garanayak M. (2019). Recommender system using item based collaborative filtering (CF) and K-means. Int. J. Knowl.-Based Intell. Eng. Syst..

[10] Roy R., Dietz L.W. (2021). TripRec - A recommender system for planning composite city trips based on travel mobility analysis. CEUR Workshop Proc..

[11] Su X. (2019). An edge intelligence empowered recommender system enabling cultural heritage applications. IEEE Trans. Ind. Inform..

[12] Huda C., Ramadhan A., Trisetyarso A., Abdurachman E., Heryadi Y. (2021). Smart tourism recommendation model: a systematic literature review. Int. J. Adv. Comput. Sci. Appl..

[13] Jin X. (2019). Motivation and involvement in adventure tourism activities: a Chinese tourists’ perspective. Asia Pac. J. Tour. Res..

[14] Harywanto G.N., Veron J.S., Suhartono D. (2021). An annotated dataset for identifying behaviour change based on five doors theory under coral bleaching phenomenon on Twitter. Data Br..

[15] Riccosan K.E.Saputra, Pratama G.D., Chowanda A. (2022). Emotion dataset from Indonesian public opinion. Data Br..

[16] Sutoyo R., Achmad S., Chowanda A., Andangsari E.W., Isa S.M. (2022). PRDECT-ID: Indonesian product reviews dataset for emotions classification tasks. Data Br..

